# A plant diterpene counteracts juvenile hormone-mediated gene regulation during *Drosophila melanogaster* larval development

**DOI:** 10.1371/journal.pone.0200706

**Published:** 2018-07-16

**Authors:** Sang Woon Shin, Jun Hyoung Jeon, Seon Ah Jeong, Ji-Ae Kim, Doo-Sang Park, Yunhee Shin, Hyun-Woo Oh

**Affiliations:** 1 Core Facility Management Center, Korea Research Institute of Bioscience and Biotechnology, Daejeon, Republic of Korea; 2 Biological Resource Center, Korea Research Institute of Bioscience and Biotechnology, Jeongeup, Republic of Korea; 3 Department of Integrative Biology, University of California, Berkley, California, United States of America; Institute of Plant Physiology and Ecology Shanghai Institutes for Biological Sciences, CHINA

## Abstract

Many plant species possess compounds with juvenile hormone disruptor (JHD) activity. In some plant species, such activity has been attributed to diterpene secondary metabolites. Plant JHD diterpenes disrupt insect development by interfering with the juvenile hormone (JH)-mediated formation of JH receptor complexes. Here, we demonstrate that a plant extract and a diterpene from *Lindera erythrocarpa* (methyl lucidone) interfere with the formation of both methoprene-tolerant (Met)/Taiman and Germ cell-expressed (GCE)/Taiman heterodimer complexes in yeast two-hybrid assays *in vitro*. In addition to the *in vitro* JHD activity, the diterpene and the plant extract from *L*. *erythrocarpa* also disrupt the development of larvae and pupae in *Drosophila melanogaster*. Comparing the transcriptomes of juvenile hormone analog (JHA, methoprene)- and JHD (methyl lucidone)-fed wandering third-instar larvae revealed a large number of genes that were coregulated by JHA and JHD. Moreover, most (83%) of the genes that were repressed by methyl lucidone were significantly activated by methoprene, indicating that JHDs and JHAs have opposing effects on the transcriptional regulation of many JH-dependent genes. Gene ontology analysis also suggested that some of the genes activated-by-JHA/repressed-by-JHD play roles in spermatogenesis. Affymetrix microarray-based analysis indicated that the expression of genes activated-by-JHA/repressed-by-JHD was testis-specific. Together, these results suggest that JH is involved in testis-specific gene expression and that plant JHD diterpenes function as JH antagonists in such JHA-mediated gene regulation.

## Introduction

Insect juvenile hormone (JH) regulates larval development and prevents premature metamorphosis [1]. Insect JH is also involved in a variety of essential physiological functions in adult insects, including reproduction, pheromone production, and cast differentiation in social insects [2], and also plays a role in regulating the development, maturation, and functioning of male accessory glands (MAGs) [3–8]. JH treatment mimics copulation-induced increases in protein synthesis in MAGs in virgin *Drosophila melanogaster* [9], and mutational studies have revealed the involvement of JH in the mating behavior of male *D*. *melanogaster* [10]. Treatment with a JH analog, hydroprene increased the expression of MAG proteins in the red flour beetle, *Tribolium castaneum*, whereas RNAi-mediated JH deficiency reduced MAG expression [11].

Methoprene-tolerant (Met) has been characterized as the JH receptor in *D*. *melanogaster*, where null mutants of the *Met* gene confer strong resistance to both JH and the JHA methoprene [12, 13], and in *Drosophila*, the germ cell-expressed (GCE) gene, which is a *Met* paralog, was characterized as a redundant JH receptor [14–17]. In contrast, other insects, except the lower dipterans, only possess a single functional *Met* gene [18]. Although *Met* null mutant flies are fully viable [12], the simultaneous loss of *Met* and *GCE* is apparently lethal during the pupal-adult transition [15, 16, 19]. In addition, the overexpression of *Met*, but not *GCE*, increases larval mortality in the absence of methoprene [20], and methoprene-fed insects die at the pharate adult stage [20, 21].

Insect Met proteins bind to JH with a high affinity and activate the transcription of JH-dependent genes [22–24]. Met, like the other members of the bHLH-PAS family of transcription factors, requires heterodimer partners (other bHLH-PAS proteins) to function appropriately [25]. The bHLH-PAS domain-containing steroid receptor coactivator (SRC) (i.e., betaFTZ-F1 interacting steroid receptor coactivator, FISC in *Aedes aegypti*, or Taiman in *D*. *melanogaster*) interacts with Met during JH-dependent gene regulation in *A*. *aegypti* [26], *T*. *castaneum* [27], and the silkworm *Bombyx mori* [23]. Met also dimerizes with the bHLH-PAS circadian clock protein Cycle (Cyc) in a JH- and circadian rhythm-dependent manner in *A*. *aegypti* [28]. In *D*. *melanogaster*, GCE was shown to bind to JH at physiological concentrations *in vitro* [17].

In previous studies, the JH-dependent heterodimer-binding properties of Met and CYC/SRC were used to develop the *in vitro* assay system to quantify the disruption caused by plant extracts and diterpenes on the JH-mediated Met-CYC/SRC heterodimer formation in the mosquito *A*. *aegypti* and the Indian meal moth *Plodia interpunctella* [29, 30]. The system involved yeast two-hybrid (Y2H) microplate simulation of pyriproxyfen-mediated *A*. *aegypti* Met/CYC binding and JH III-mediated *P*. *interpunctella* Met/SRC binding. Use of the *A*. *aegypti* Y2H screening system revealed that methyl linderone, a diterpene from *Lindera erythrocarpa*, possessed strong JH disruptor (JHD) activity and is effective in killing the mosquito larvae [29]. In addition, a conifer diterpene resin acid, 7-oxodehydroabietic acid, from *Pinus densiflora* strongly interfered with JH-mediated *P*. *interpunctella* Met-SRC binding and disrupted the larval development in *P*. *interpunctella* [30].

In this study, *D*. *melanogaster* Y2H screening systems for Met-Taiman and SRC-Taiman binding were constructed. In these *in vitro* systems, Met-Taiman binding was induced by JH III or JHAs, whereas GCE-Taiman binding is constitutive and occurs independent of JH or JHAs. Both, the Met-Taiman and GCE-Taiman binding can be disrupted by plant extracts or JHD diterpenes, especially methyl lucidone. Accordingly, both *L*. *erythrocarpa* extracts and methyl lucidone strongly blocked the larval and pupal development of *D*. *melanogaster*, thereby preventing the formation of pupae and emergence of adults. In addition, the comparison of transcriptomes from methoprene- and methyl lucidone-fed wandering third-instar larvae revealed that many testis-specific genes were up-regulated by JHA and down-regulated by JHD, indicating that JHD counteracts the role of JHA during the JH-mediated regulation of testis-specific genes.

## Materials and methods

### Chemicals

JH-III, methoprene, and pyriproxyfen were purchased from Sigma-Aldrich (St. Louis, MO, USA), and the three plant diterpenes (kanakugiol, methyl linderone, and methyl lucidone) were isolated from *L*. *erythrocarpa* as described previously [29]. Each reagent was prepared as a stock solution in dimethyl sulfoxide (DMSO) for the Y2H assays, and in ethanol for the larval development and RNA-seq tests.

### Construction of *in vitro* JH/JHD assay systems

Two bait plasmids were constructed by cloning Met and GCE cDNAs with full-length open reading frames (ORFs) in the GAL4 DNA-binding domain of the pGBKT7 vector (Clontech, Mountain View, CA, USA). A prey plasmid was constructed by cloning the GAL4-AD fusion plasmid with a partial Taiman cDNA in the pGADT7 vector (Clontech), and using the following primers:

*D*. *melanogaster* Met forward: 5′- GCTACATATGGCAGCACCAGAGACGG -3′,
*D*. *melanogaster* Met reverse: 5′- GCTAGAATTCTCATCGCAGCGTGCTGGTC -3′,
*D*. *melanogaster* GCE forward: 5′- GCTACATATGGAGGGTGCCAGTCGCAGC -3′,
*D*. *melanogaster* GCE reverse: 5′- GCTAGTCGACAACTATTGCAGTCGTACAT -3′,
*D*. *melanogaster* Taiman forward: 5′- CATATGTCAATTGCTGCAGCCGAAAATG -3′,
*D*. *melanogaster* Taiman reverse: 5′- GCTAGAATTCCAAGCCCAGTCCTCCACTG -3′,

### Yeast β-galactosidase assay

For the Y2H binding assay, Y187 yeast cells were transformed with both, the bait (Met or GCE) and prey (Taiman) plasmids. The transformed Y187 cells were incubated at 30°C in SD-Leu/-Trp double-dropout medium until reaching an OD_600_ of 0.3–0.4. The yeast cells were harvested by centrifugation and suspended in twice the volume of culture media. The cells were incubated for an additional 2 h, and 100 μL aliquots of the resulting cell cultures (OD_600_ = 0.2–0.3) were transferred to individual wells of 96-well plates. JH III and each JHA were added to the growth media to concentrations of 0.01, 0.1, 1, and 10 ppm, and the cells were incubated for an additional 3 h before being assayed using a yeast β-galactosidase assay kit (Thermo Scientific, Waltham, MA, USA). The assay reaction mixtures were incubated at 30°C for 16 h and then subject to OD_420_ measurement.

### JHD activity assay of plant extracts and JHD diterpenes

The transformed Y187 cells (Met-Taiman or GCE-Taiman) were grown as described in the preceding section. For the Met-Taiman JHD assay, 100 μL of grown yeast cells (OD_600_ = 0.2–0.3) were treated with 0.1 ppm pyriproxyfen along with 100 ppm of plant extract (methanolic extracts prepared from the Korean Plant Extracts Bank, Daejeon, Korea) or the corresponding concentrations of JHD compounds in 96-well plates. Both, a positive control (0.1 ppm pyriproxyfen and 10 ppm methyl linderone) and negative control (0.1 ppm pyriproxyfen and DMSO) were included for each plate. The cells were incubated for an additional 3 h and were subjected to the quantitative β-galactosidase assay. The OD_420_ values obtained were normalized to an arbitrary unit of JHD activity. Methyl linderone was used as a positive control, and the level of binding interference by 10 ppm methyl linderone was calculated as a single arbitrary unit of JHD activity. The averages of triplicate experiments were used as the specific JHD activity of each plant extract:

$$\text{A}=\frac{{\text{OD}}_{420}\;\text{Control}-{\text{OD}}_{420}\;\text{PE}}{{\text{OD}}_{420}\;\text{Control}-{\text{OD}}_{420}\;\text{ML}10},$$

where *A* represents the JHD activity (if *A* < 0, then *A* = 0), the *Control* group was treated with a corresponding concentration of JH, the *PE* group was treated with 0.1 ppm pyriproxyfen and 100 ppm plant extract, and the *ML10* group was treated with 0.1 ppm pyriproxyfen and 10 ppm methyl linderone. The same protocol was used for the GCE-Taiman JHD assay, except for the addition of 0.1 ppm pyriproxyfen.

### Bioassays of plant extracts and diterpenes to evaluate effects on *D*. *melanogaster* development

Twenty male and 20 female flies were added to individual vials, each containing 3 g artificial diet mixed either with a plant extract or a diterpene. The 10% stock solution of each plant extract or each diterpene was prepared by dissolving 100 mg/ml concentration in ethanol. The 10% solution was further diluted with ethanol to yield a 5x solution of the corresponding concentration of the plant extracts. For example, in order to prepare the diet with 0.5% plant extract, 1 volume of 10% stock solution was diluted with 3 volumes of ethanol to yield 2.5% solution. From this, 1 volume of 2.5% solution was further mixed with 4 volumes of molten *Drosophila* diet. For control (0% diet), 1 volume of ethanol was mixed with 4 volumes of molten *Drosophila* diet. The mixed diets were stored at room temperature overnight to solidify and for evaporation of ethanol. After 2 d of oviposition (200–300 eggs per vial), the adult flies were removed from the vials. After 2 weeks, the third-instar larvae, pupae, and emerging adults were counted. After counting, the wandering third-instar larvae and pupae were further cultured in order to record the number of adults emerging within 3 weeks.

### Gene expression profiling using Illumina RNA sequencing

Twenty male and 20 female adult flies were added to individual vials, each containing 3 g artificial diet mixed with either 0.5% methyl lucidone (w/v), 0.05% methoprene (w/v), or 0.5% ethanol (w/v), as a control. After 2 d of oviposition, the adult flies were removed from the vials, and the laid eggs were allowed to develop. After 7 d, the wandering third-instar larvae were collected from each vial, the total RNA was isolated. This RNA was subjected to Illumina RNA-sequencing (S1 Table, independently triplicated). Average FPKM values of control samples, differences in average FPKM values between the control and the treatment samples, and significance of differences (q-values < 0.01, Benjamini-Hochberg) were calculated using Tophat, Cufflinks, and Cuffdiff [31]. Among a total of 17,450 *D*. *melanogaster* genes annotated by the Berkeley Drosophila Genome Project (BDGP, Release 6), 10,000 abundantly expressed protein-coding genes were selected for further analyses (S2 Table). Among these 10,000 genes, those with significantly different expression levels in the control and treatment groups were selected. A total of 696 were significantly activated by JHA and significantly repressed by JHD (JHA↑JHD↓), whereas 337 genes were significantly repressed by JHA and significantly activated by JHD (JHA↓JHD↑), 278 genes significantly activated by both JHA and JHD (JHA↑JHD↑), and 320 genes were significantly repressed by both JHA and JHD (JHA↓JHD↓).

### GO analysis and Affymetrix microarray-based atlas of gene expression analyses

Our gene lists (JHA↑JHD↓H JHA↑JHD↑H JHA↑JHD↑H and JHA↓JHD↓) were applied to the FlyMine database [43] to identify enriched GO categories [44] and to characterize tissue-specific expression [45]. In addition, the Evolutionary Genealogy of Genes: Non-supervised Orthologous Groups (eggNOG) database, which contains orthologous groups constructed from the Smith-Waterman alignments through identification of reciprocal best matches and triangular linkage clustering [46], was used to specify the overrepresented gene functions. The *D*. *melanogaster* eggNOG 4.0 database was adjusted to include insect-specific gene lineages, such as the antimicrobial peptides and cuticular proteins (S1 Table).

### RNA extraction, primers, and qPCR analysis

An RNeasy kit (Qiagen, Hilden, Germany) was used to extract total RNA from the second-, early third-, and wandering third-instar larvae that were fed with ethanol (control)-, methoprene (JHA)-, or methyl lucidone (JHD)-supplemented diet. The cDNAs were synthesized for qPCR using a Tetro cDNA Synthesis Kit (Bioline, London, United Kingdom) and 1 μg RNA, as estimated using a NanoDrop ND-1000 Spectrophotometer (Thermo Scientific).

Primer pairs were designed for the target genes, namely *ocnus* (*ocn*), *janus B* (*janB*), *Glycine N-methyltransferase* (*Gnmt*), *Odorant-binding protein 99b* (*obp99b*), *Sperm-Leucyl aminopeptidase 1* (*S-lap1*), *Male-specific RNA 87F* (*Mst87F*), *don juan* (*dj*), and *don juan like* (d*jl*), using Primer 3 [47, 48]:

*D*. *melanogaster* ocn forward: 5′- CGCCCTTTTGATAAATGTTC -3′,
*D*. *melanogaster* ocn reverse: 5′- CGCAAATTCCAATTTTGTCC -3′,
*D*. *melanogaster* janB forward: 5′- CTCGCACTAAACCTTTTCGG -3′,
*D*. *melanogaster* janB reverse: 5′- GTTATCTTGACCCGGGGAAC -3′,
*D*. *melanogaster* Gnmt forward: 5′- CCGTAGATGCCTCTGATAAG -3′,
*D*. *melanogaster* Gnmt reverse: 5′- CAAGTGGGCAAAGGAGTTGC -3′,
*D*. *melanogaster* obp99b reverse: 5′- ATTTCACTTTGATTGCTTCG -3′,
*D*. *melanogaster* obp99b reverse: 5′- GTGCGATAGTTGGTCAGATC -3′,
*D*. *melanogaster* S-lap1 reverse: 5′- GAAGGAGCTCTTCAACATGC -3′,
*D*. *melanogaster* S-lap1 reverse: 5′- CCTCAATGGTCTTGCCATTC -3′,
*D*. *melanogaster* Mst87F reverse: 5′- CGAATTAATCATGTGCTGCG -3′,
*D*. *melanogaster* Mst87F reverse: 5′- CCTATCGTCTTGGTGTTCTG -3′,
*D*. *melanogaster* dj reverse: 5′- ACGGCCTCACCACATCAATG -3′,
*D*. *melanogaster* dj reverse: 5′- CTAACAGTCTGTTGCTGCTC -3′,
*D*. *melanogaster* djl reverse: 5′- TTCTCAAGCCCGAAAGCCAG -3′,
*D*. *melanogaster* djl reverse: 5′- CATCGCCATCATTAGCCTGC -3′,

The qPCR was performed using RealFAST SYBR kit (Geneer, Daejeon, Korea) in 48-well plates on the Eco Real-Time PCR System (Illumina, San Diego, CA, USA). The following two-step thermal cycler program was used for all runs: 95°C for 3 min; 40 cycles of 95°C for 5 s and 60°C for 20 s; and a final melting curve analysis spanning 95°C for 15 s, 55°C for 15 s, and 95°C for 15 s. Eco Manager Software (Illumina) was used to validate amplification efficiency and specificity.

## Results

### Plant species possess *in vitro* JHD activity against *D*. *melanogaster* Met and GCE

**Fig 1:**
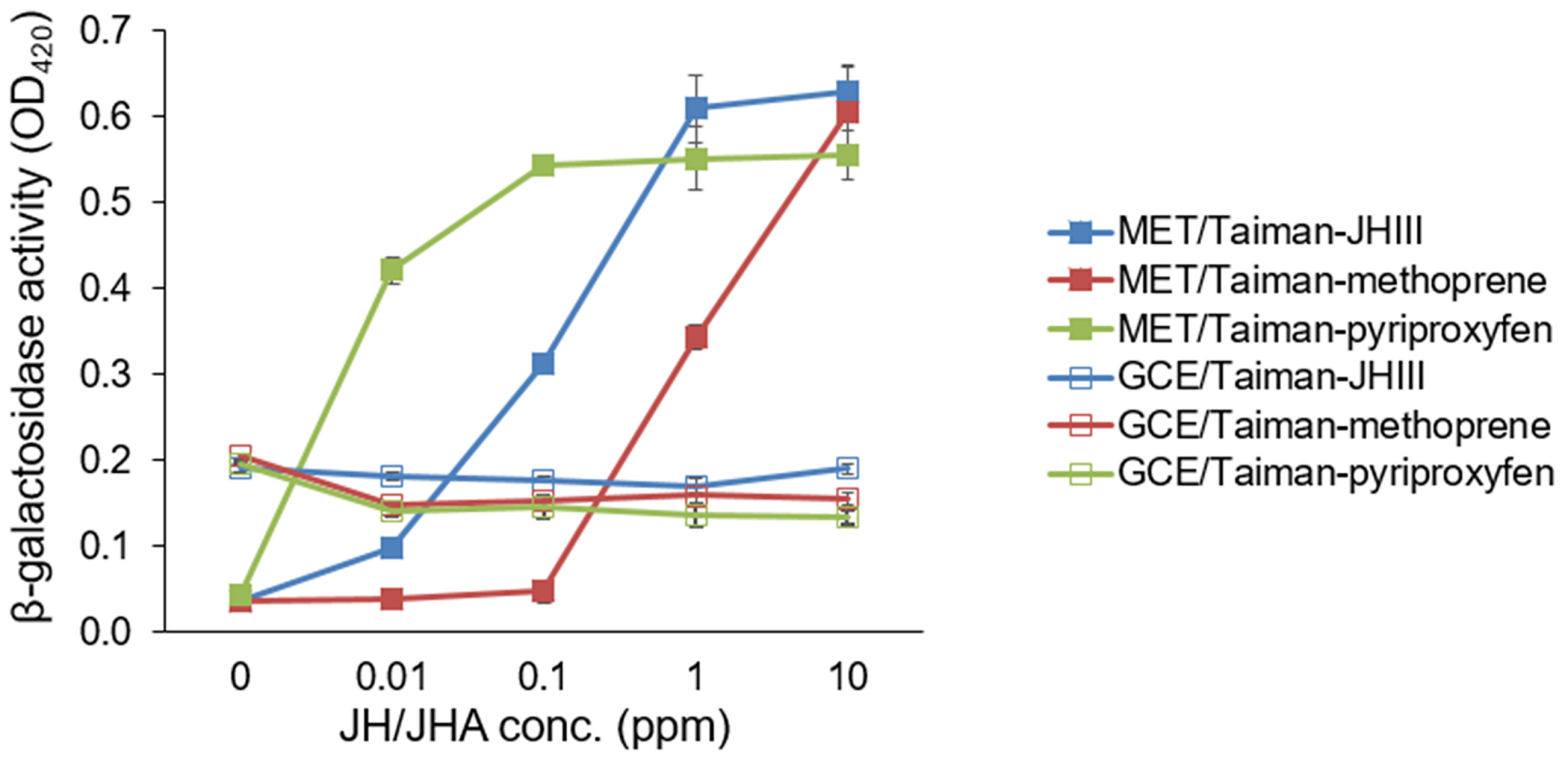
Microplate simulation of juvenile hormone III (JH III)- or juvenile hormone analog (JHA)-dependent Met-Taiman or GCE-Taiman binding. Yeast Y197 cells were transformed using yeast two-hybrid (Y2H) plasmids that contained Met-Taiman or GCE-Taiman and were incubated with different concentrations of JH III or JHAs. The JH III/JHA-mediated binding of Met-Taiman and GCE-Taiman was measured by absorbance values at 420 nm (OD_420_) after assaying β-galactosidase activity. Values and error bars indicate means ± SD (n = 3).

The cDNAs encoding full open reading frames (ORFs) of *Met* and *GCE* from *D*. *melanogaster* were introduced in a yeast two-hybrid (Y2H) bait plasmid, whereas the cDNA encoding a partial ORF of the *D*. *melanogaster* Taiman gene was introduced in a Y2H prey plasmid. In this Y2H assay, the heterodimer binding of Met-Taiman occurred in the presence of JH III or the JHAs methoprene and pyriproxyfen in a concentration-dependent manner (Fig 1). Meanwhile, GCE-Taiman binding occurred constitutively, i.e., in the absence of JH III or JHAs, and was not enhanced by the addition of JH III or JHAs (Fig 1).

**Fig 2:**
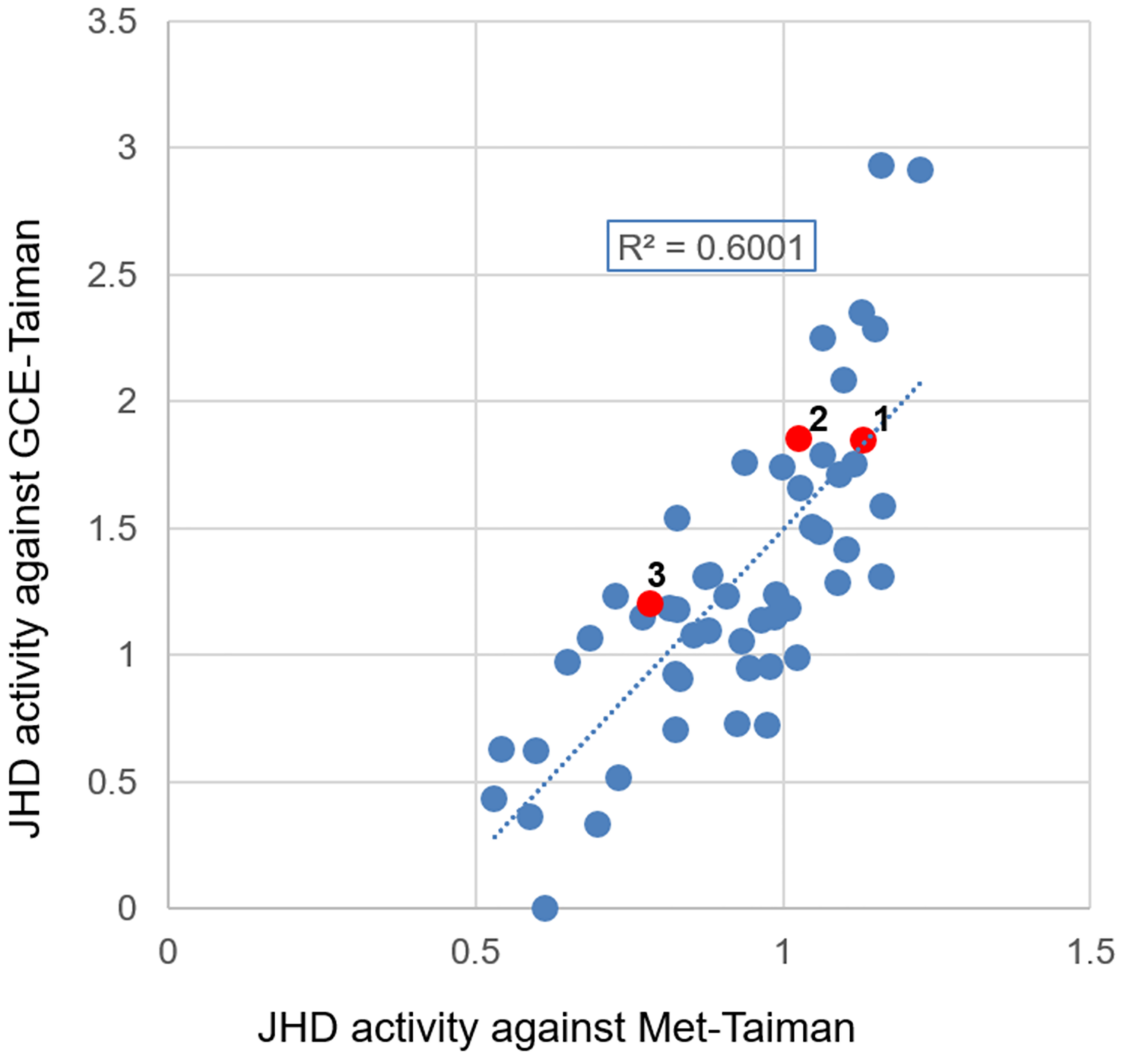
Juvenile hormone disruptor (JHD) activity of 53 plant species against Met-Taiman and GCE-Taiman binding. The JHD activities of 53 high-JHD plant species against Met-Taiman and GCE-Taiman binding were calculated as the average JHD activity of four independent experiments. Values in the rectangular box indicate the coefficients of determination (R^2^) for JHD activity of Met-Taiman and GCE -Taiman binding. 1, *Lindera erythrocarpa*; 2, *Pinus densiflora*; 3, *Solidago serotina*.

The *in vitro* disrupting activity of 53 plant extracts that were previously reported to exhibit relatively strong *in vitro* JHD activity against JHA (pyriproxyfen)-mediated *A*. *aegypti* Met-CYC binding and to possess *in vivo* larvicidal activity against the mosquito *A*. *aegypti* [29] were tested against pyriproxyfen-induced Met-Taiman binding or constitutive GCE-Taiman binding (Fig 2). In general, the plant extracts disrupted both Met-Taiman and GCE-Taiman binding (Fig 2, R^2^ = 0.6001). These results demonstrate that GCE-Taiman binding occurs independently of JH/JHA and that the binding can be disrupted by plant extracts with *in vitro* JHD activity, which suggests that plant JHDs may be non-structural antagonists of JH/JHA and do not directly compete with JH/JHA in the JH-binding pocket of JH receptor complexes.

### *L*. *erythrocarpa* extract and methyl lucidone block *D*. *melanogaster* larval development

**Fig 3:**
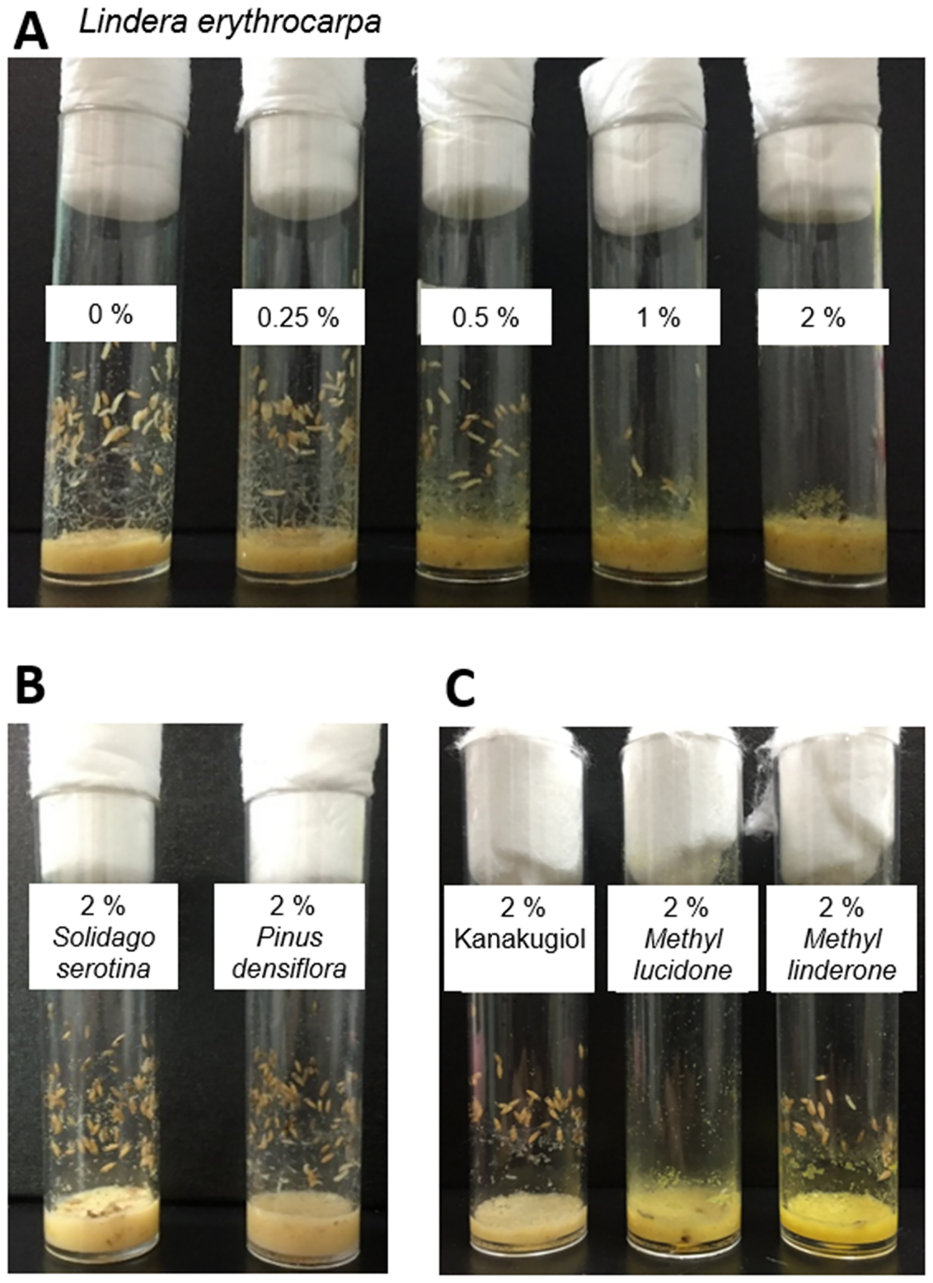
Effect of *Lindera erythrocarpa*, *Solidago serotina*, and *Pinus densiflora* extracts and three juvenile hormone disruptor (JHD) diterpenes from *L*. *erythrocarpa* on the larval development of *Drosophila melanogaster*. (A) Effect of *L*. *erythrocarpa* on the development of *D*. *melanogaster* larvae at a concentration-dependent manner. (B) Effect of 2% (w/v) *S*. *serotina* and *P*. *densiflora* extracts on the development of *D*. *melanogaster* larvae. (C) Effect of 2% (w/v) methyl lucidone, a JHD diterpene from *L*. *erythrocarpa*, on the development of *D*. *melanogaster* larvae.

**Fig 4:**
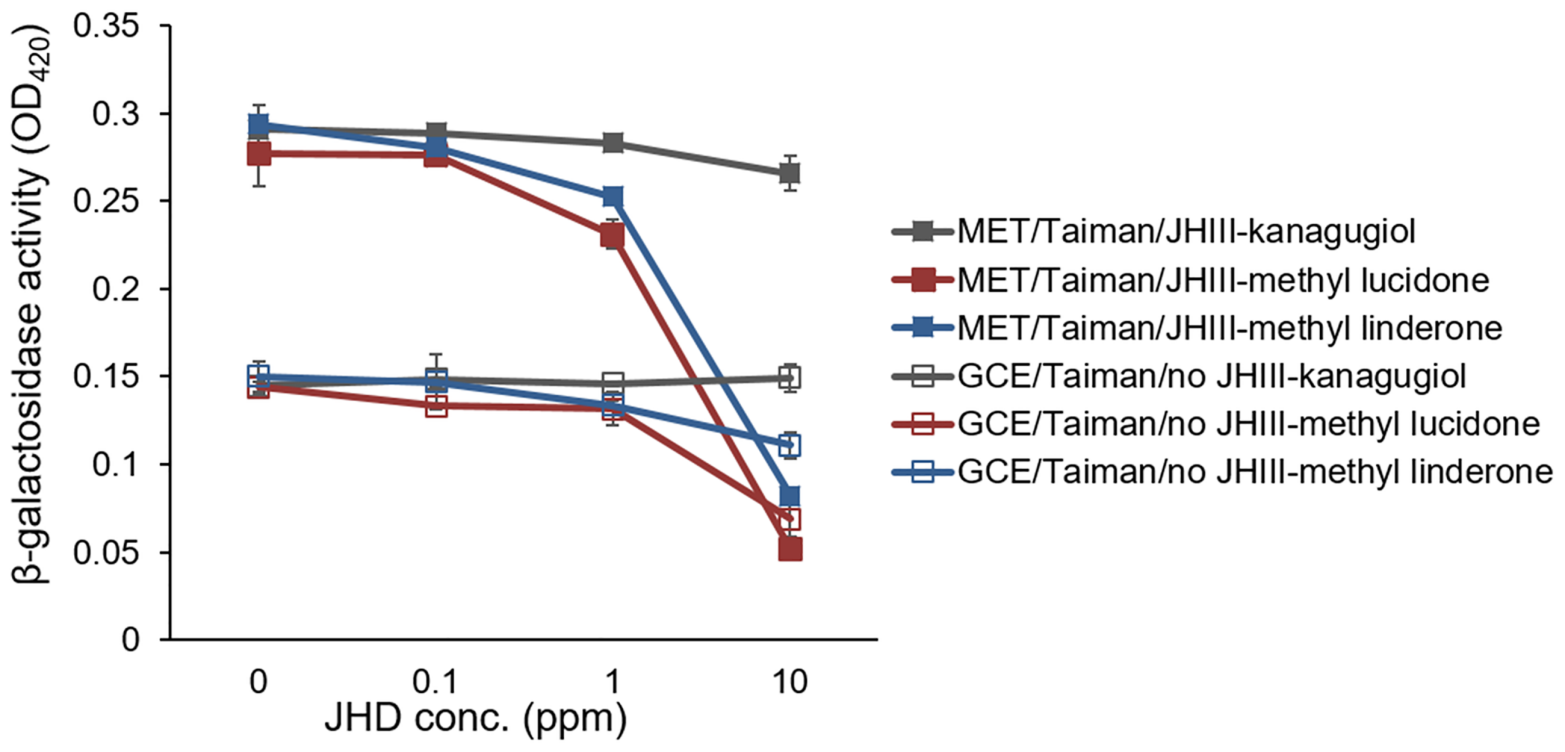
Juvenile hormone disruptor (JHD) activities of three *Lindera erythrocarpa* diterpenes against Met-Taiman and GCE-Taiman binding. Dose-dependent inhibition of pyriproxyfen-mediated Met-Taiman binding or constitutive GCE-Taiman binding was observed for two *L*. *erythrocarpa* JHD diterpenes, i.e., methyl lucidone and methyl linderone. Kanakugiol did not significantly disrupt either Met-Taiman or GCE-Taiman binding. Values and error bars indicate means ± SD (n = 4).

When extracts of *L*. *erythrocarpa*, *S*. *serotina*, and *P*. *densiflora*, previously reported to disrupt larval development of the mosquito or the moth [29, 30], were added to the diets of *D*. *melanogaster* larvae, the *L*. *erythrocarpa* extract strongly blocked larval development in a concentration-dependent manner (Fig 3A). Indeed, the diet containing 2% (w/w) *L*. *erythrocarpa* extract reduced the larval numbers and prevented pupal development (Fig 3A). However, the *S*. *serotine* and *P*. *densiflora* extracts (2%, w/w) failed to significantly affect larval development (Fig 3B). When JHD diterpenes from *L*. *erythrocarpa* (i.e., kanakugiol, methyl lucidone, and methyl linderone) were added to the diets, the eggs cultured in the 2% methyl lucidone-containing diet completely failed to develop as pupae or adults (Fig 3C). Of the three JHD diterpenes, methyl lucidone most strongly interfered with both JHA-mediated Met-Taiman binding and constitutive GCE-Taiman binding (Fig 4).

### Effects of JHD and JHA treatments on the *Drosophila* transcriptome

**Fig 5:**
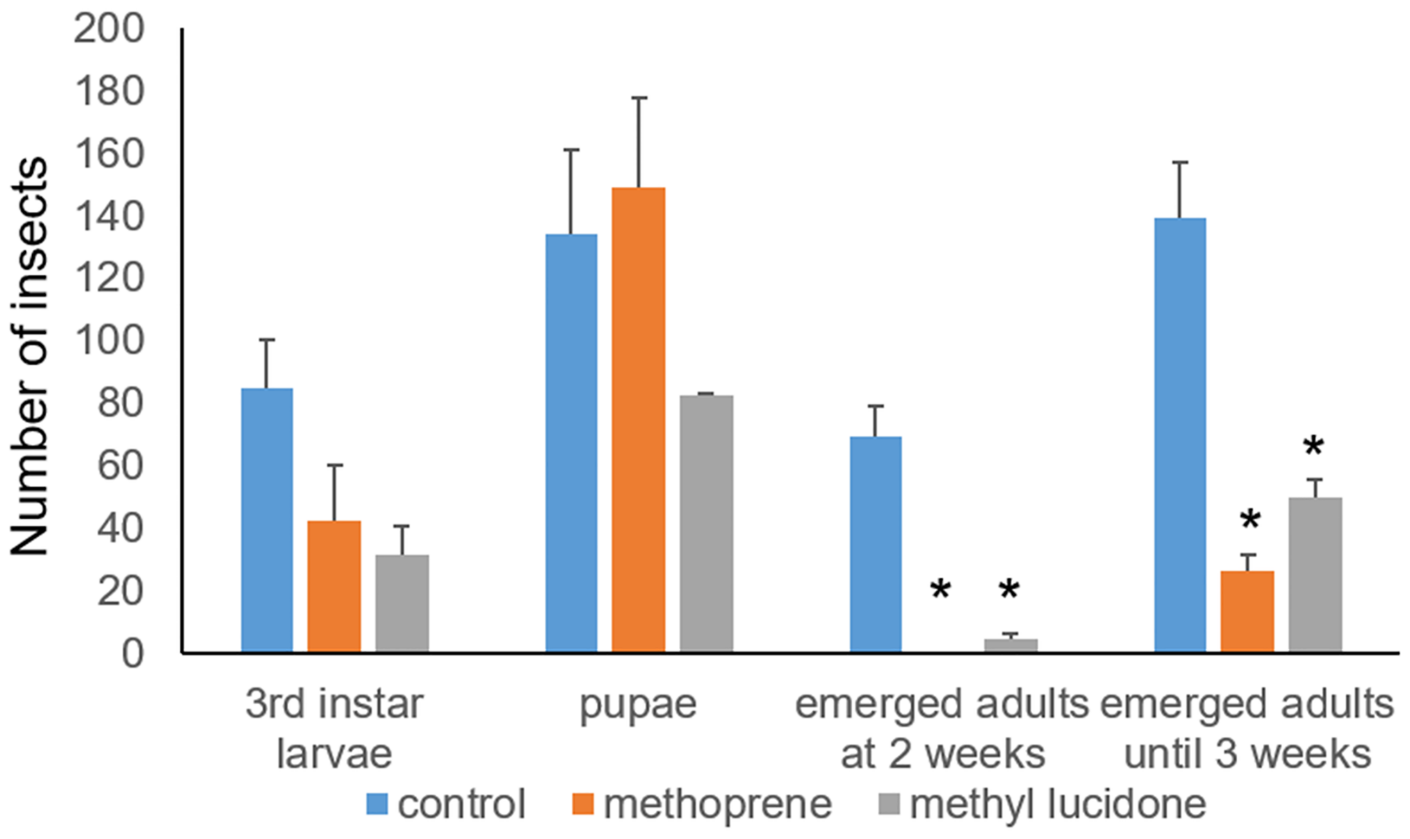
Larval and pupal development of *Drosophila melanogaster* fed sublethal doses of juvenile hormone analog (JHA) or disruptor (JHD). Values and error bars indicate means ± SD (n = 3). *, p < 0.05 (*t*-test).

In order to investigate the role of methyl lucidone on JH-mediated gene regulation, the transcriptomes of the wandering third-instar larvae that were hatched and cultured in a methyl lucidone (0.5%, w/w)-supplemented diet were compared to those larvae that were cultured in either ethanol (control)- or JHA (methoprene, 0.05%, w/w)-supplemented diets. At these sublethal concentrations, the JHA or JHD treatment significantly reduced the number of developed adults (Fig 5).

The abundance of specific transcripts in the control, JHA-treated, and JHD-treated samples were compared using RNA-seq analysis pipelines, such as TopHat2, Cufflinks, and Cuffdiff [31]. The 10,000 protein-coding genes that were most abundant in the transcriptomes of the control samples were selected from among 17,450 annotated genes by the Berkeley *Drosophila* Genome Project Release 6 (BDGP6) for further analyses (S1 Table). The differentially expressed transcripts (q < 0.01 after false discovery rate correction) were identified by comparing the transcription levels of the control transcriptome to those of the JHA-treatment and JHD-treatment transcriptomes. As a result, the differentially-expressed genes (DIGs) were categorized into four groups: JHA↑JHD↓ (genes significantly activated by JHA and significantly repressed by JHD; S2 Table), JHA↓JHD↑ (genes significantly repressed by JHA and significantly activated by JHD; S3 Table), JHA↑JHD↑ (genes significantly activated by both JHA and JHD; S4 Table), and JHA↓JHD↓ (genes significantly repressed by both JHA and JHD; S5 Table).

**Fig 6:**
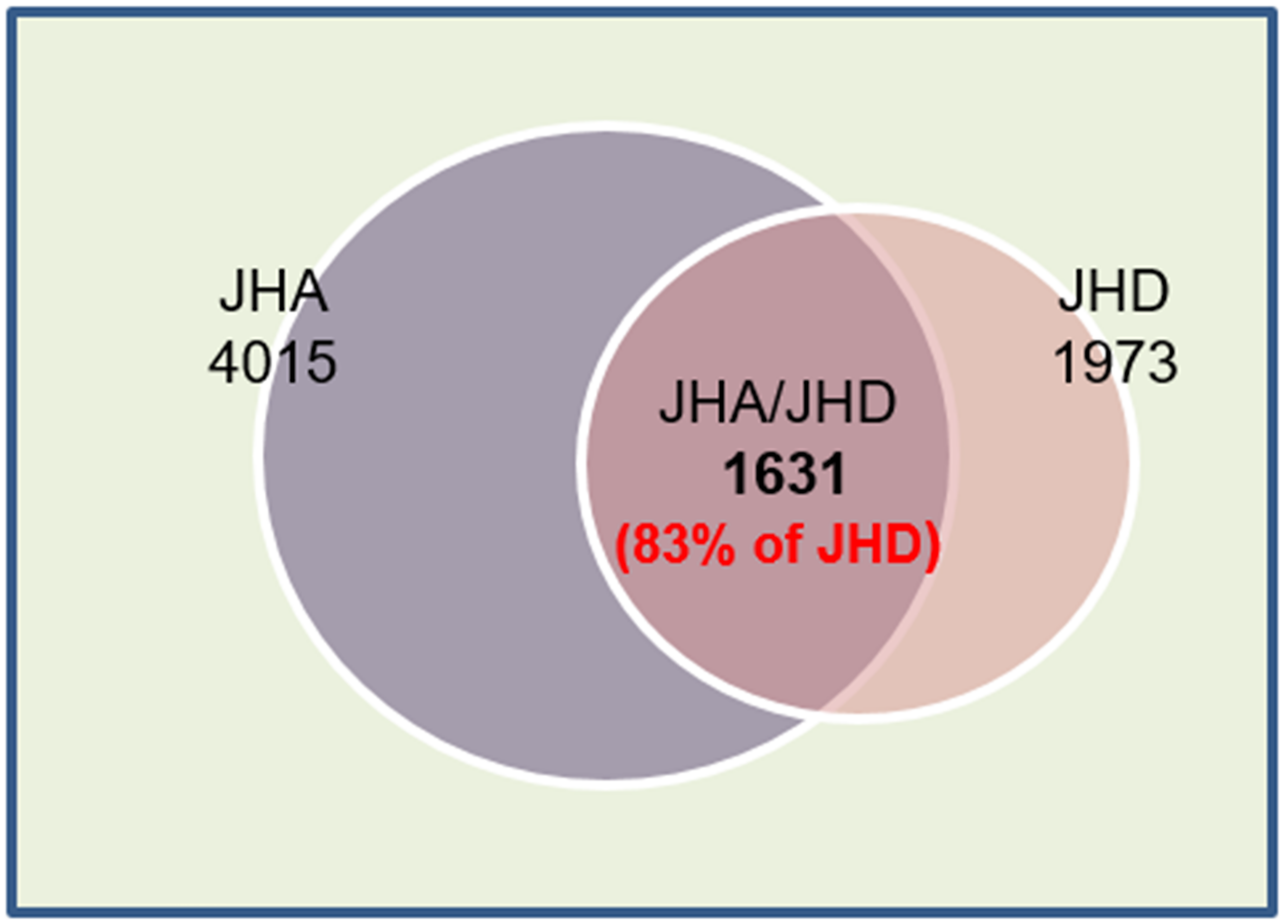
Illumina RNA-seq transcriptome analysis of juvenile hormone analog (JHA, methoprene)- and disruptor (JHD, methyl lucidone)-treated *Drosophila melanogaster* larvae. JHA, genes significantly affected by JHA; JHD, genes significantly affected by JHD; JHA/JHD, genes significantly affected by both JHA and JHD; JHA↑, genes significantly activated by JHA; JHD↓, genes significantly repressed by JHD; JHA↑JHD↓, genes significantly activated by JHA and significantly repressed by JHD.

**Fig 7:**
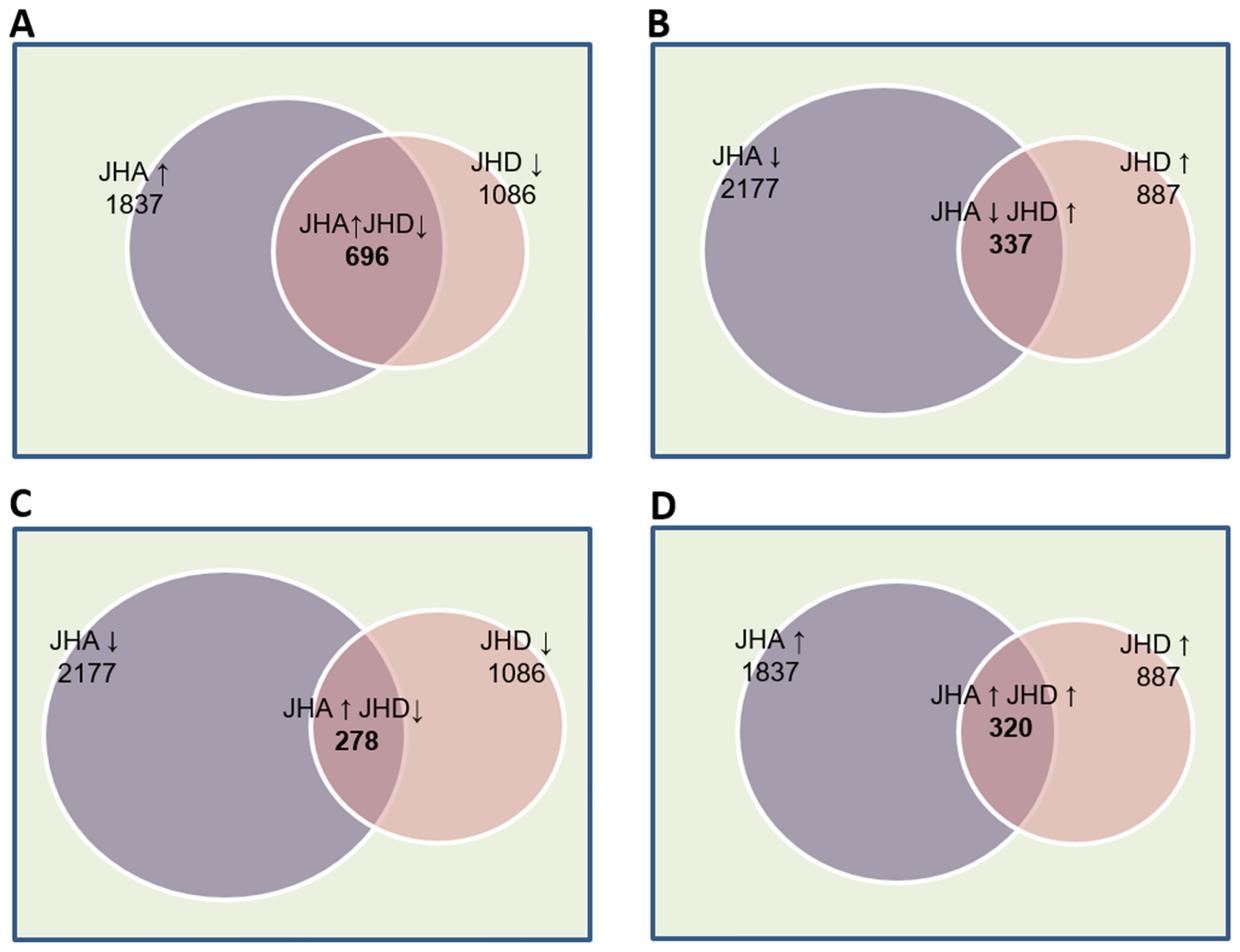
Transcriptome analysis of genes that were differentially expressed in juvenile hormone analog (JHA, methoprene)- and disruptor (JHD, methyl lucidone)-treated *Drosophila melanogaster* larvae. JHA↑, genes significantly activated by JHA treatment; JHA↓, genes significantly repressed by JHA treatment; JHD↑, genes significantly activated by JHD treatment; JHD↓, genes significantly repressed by JHD treatment. (A) JHA↑JHD↓, genes significantly activated by JHA and significantly repressed by JHD; (B) JHA↓JHD↑, genes significantly repressed by JHA and significantly activated by JHD; (C) JHA↑JHD↑, genes significantly activated by both JHA and JHD; and (D) JHA↓JHD↓, genes significantly repressed by both JHA and JHD.

Most (n = 1631, 83%) of the DIGs from the JHD-treated group overlapped with those from the JHA-treated group (Fig 6), and among the DIGs from both the JHA- and JHD-treated groups, more genes belong to the JHA↑JHD↓ group of DIGs (Fig 7A; 696 genes), than to the JHA↓JHD↑ (337 genes; Fig 7B), JHA↑JHD↑ (278 genes; Fig 7C), or JHA↓JHD↓ group of genes (320 genes; Fig 7D). In particular, 64% of the JHD-repressed (JHD↓) genes exhibited the opposite responses as those activated by JHA (JHA↑), which indicated that JHD and JHA have opposite effects on the regulation of many JH-dependent genes (Fig 7A).

### Ontology analysis of genes affected by both JHA and JHD

By applying each DIG group to the FlyMine, an integrated database for *Drosophila* (www.flymine.org), gene ontology (GO) enrichment of the four DIG groups (JHA↑JHD↓, JHA↓JHD↑, JHA↑JHD↑, and JHA↓JHD↓) was analyzed (S9–S11 Tables). A key representative GO group was indicated for each DIG group after removing the redundant GO groups (Table 1): spermatogenesis for the JHA↑JHD↓ group, chitin metabolic process for the JHA↓JHD↑ group, response to external stimulus, including defense responses to bacteria and other organisms, for the JHA↑JHD↑ group, and chitin-based cuticle development for the JHA↓JHD↓ group.

**Table 1:** Overrepresented gene ontology groups among juvenile hormone (JH)-regulated genes.

Gene group	A key overrepresented Gene Ontology group	number of genes	p-value (Holm_Bonferroni)	GO terms
**JHA↑JHD↓**	spermatogenesis	36	1.46E-08	GO:0007283
**JHA↓JHD↑**	chitin metabolic process	32	3.48E-22	GO:0006030
**JHA↑JHD↑**	response to external stimulus	53	2.4E-06	GO:0009605
**JHA↓JHD↓**	chitin-based cuticle development	15	0.011321	GO:0040003

**Fig 8:**
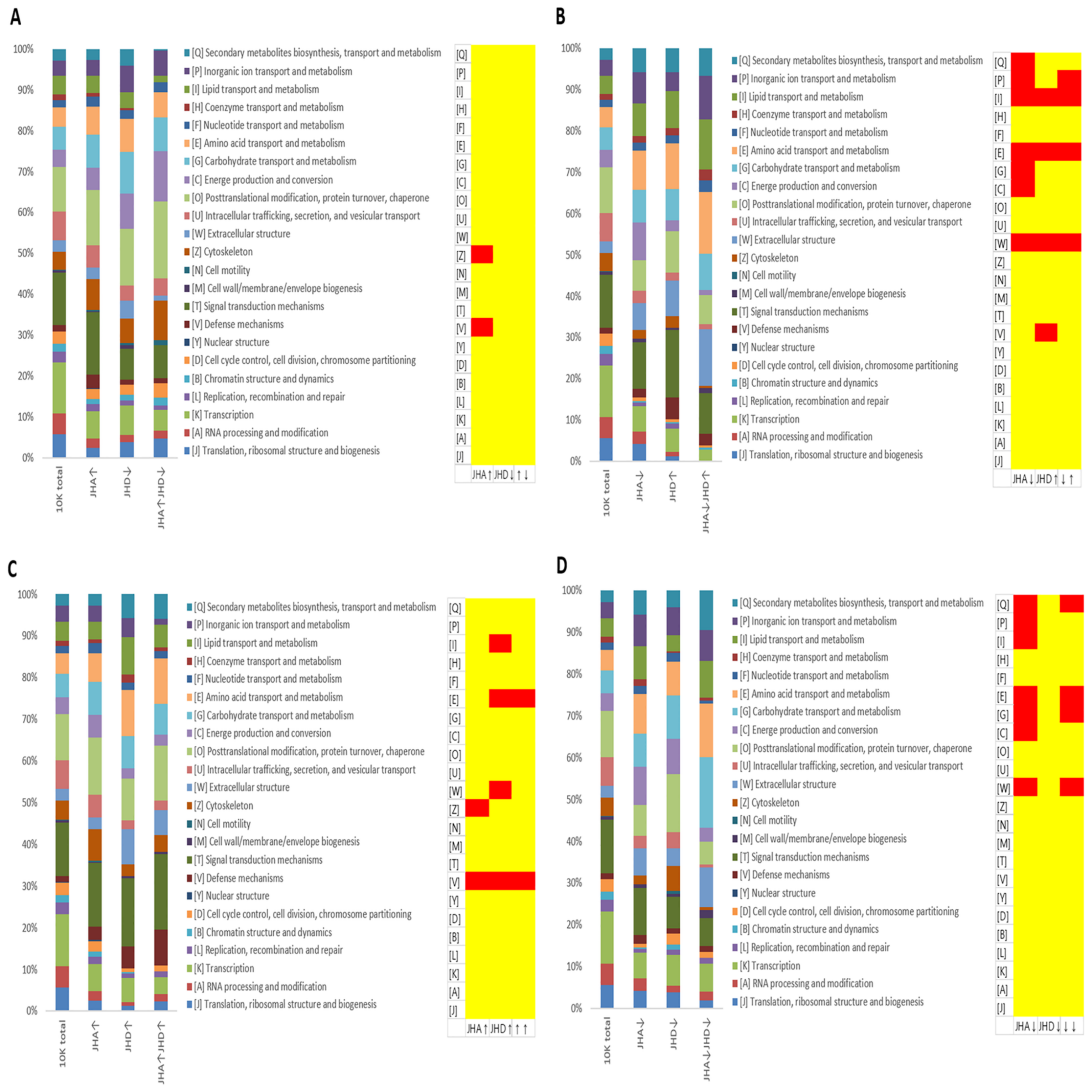
Gene ontology analysis of gene cohorts that were differentially expressed in juvenile hormone analog (JHA)- and disruptor (JHD)-fed *D*. *melanogaster* larvae. Red indicates ontology groups with significant overrepresentation (P<0.01 in a hypergeometric distribution). The functional groups with corresponding abbreviations and colors are indicated. (A) JHA↑JHD↓, genes significantly activated by JHA and significantly repressed by JHD; (B) JHA↓JHD↑, genes significantly repressed by JHA and significantly activated by JHD; (C) JHA↑JHD↑, genes significantly activated by both JHA and JHD; and (D) JHA↓JHD↓, genes significantly repressed by both JHA and JHD.

Significantly overrepresented orthologous groups (OGs) were also identified for each DIG group, using the eggNOG database. The significantly overrepresented (P < 0.01 in a hypergeometric distribution) OGs were: none for the JHA↑JHD↓ group (Fig 8A), inorganic ion transport and metabolism (P), lipid transport and metabolism (I), amino acid transport and metabolism (E), and extracellular skeleton (W) for the JHA↓JHD↑ group (Fig 8B), amino acid transport and metabolism (E) and defense mechanisms (V) for the JHA↑JHD↑ group (Fig 8C), and secondary metabolites biosynthesis, transport and metabolism (Q), amino acid transport and metabolism (E), carbohydrate transport and metabolism (G), and extracellular structure (W) for the JHA↓JHD↓ group (Fig 8D).

### JHA activation and JHD repression of testis-specific genes

**Fig 9:**
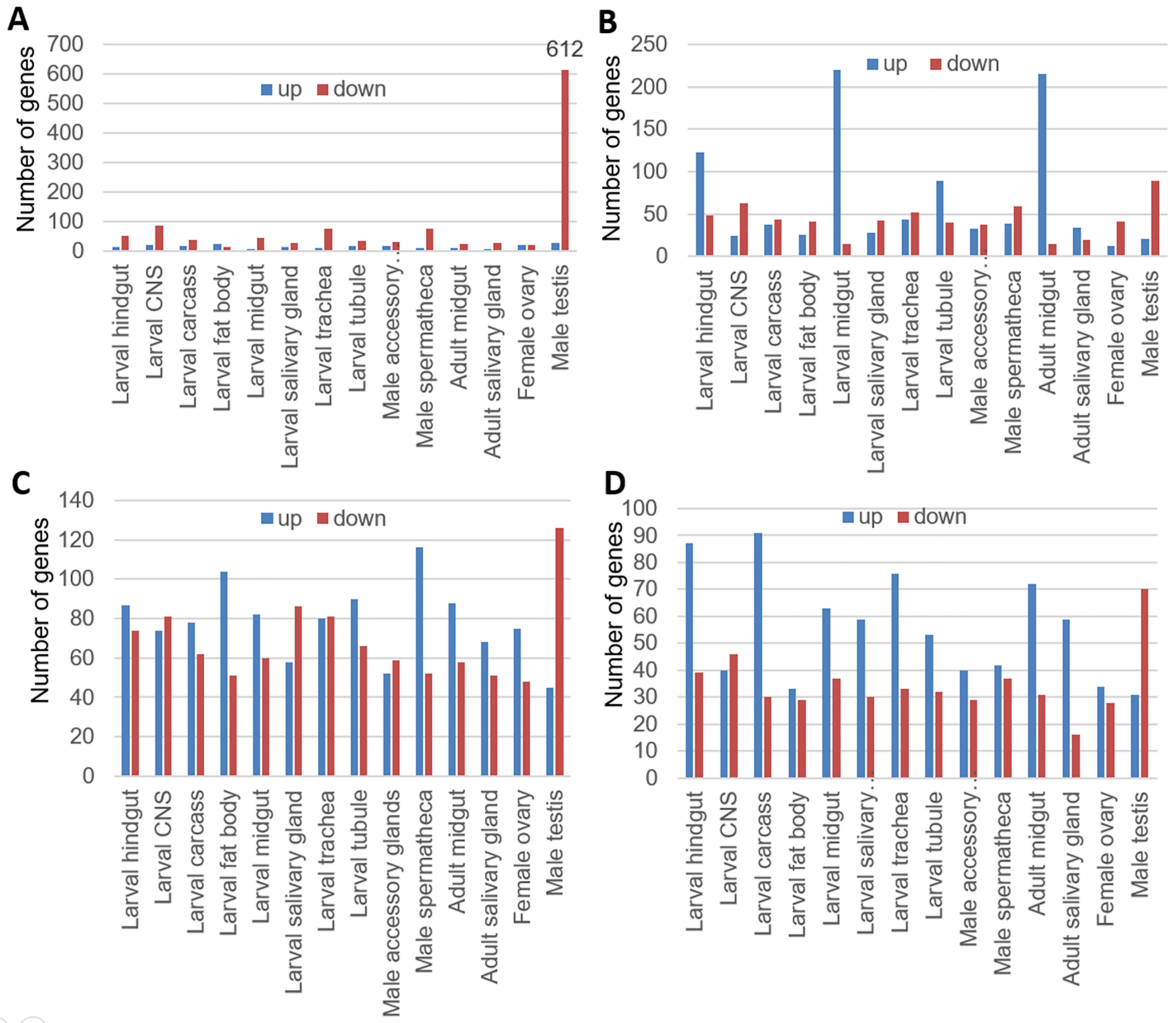
Tissue-specificity of genes that were differentially expressed in juvenile hormone analog (JHA)- and disruptor (JHD)-fed *D*. *melanogaster* larvae. The genes significantly activated by JHA and significantly repressed by JHD (S2 Table) were applied to gene expression database constructed using Affymetrix microarray results to characterize tissue-specific expression. (A) Tissue-specificity of JHA↑JHD↓ genes which were significantly activated by JHA and significantly repressed by JHD; (B) Tissue-specificity of JHA↓JHD↑ genes which were significantly repressed by JHA and significantly activated by JHD; (C) Tissue-specificity of JHA↑JHD↑ genes which were significantly activated by both JHA and JHD; and (D) Tissue-specificity of JHA↓JHD↓ genes which were significantly repressed by both JHA and JHD.

A spermatogenesis GO group was identified as a key GO group overrepresented in JHA↑JHD↓ genes (Table 1 and S6 Table). Moreover, almost all of the JHA↑JHD↓ genes (612 of 696) were upregulated in the male testis tissue (Fig 9A), when the JHA↑JHD↓ genes were submitted to the FlyMine database of Affymetrix microarray-based atlas of gene expression in larval and adult tissues [32]. The tissue-specificity of other three DIG groups (JHA↓JHD↑, JHA↑JHD↑, and JHA↓JHD↓) are also indicated (Fig 9B–9D).

**Fig 10:**
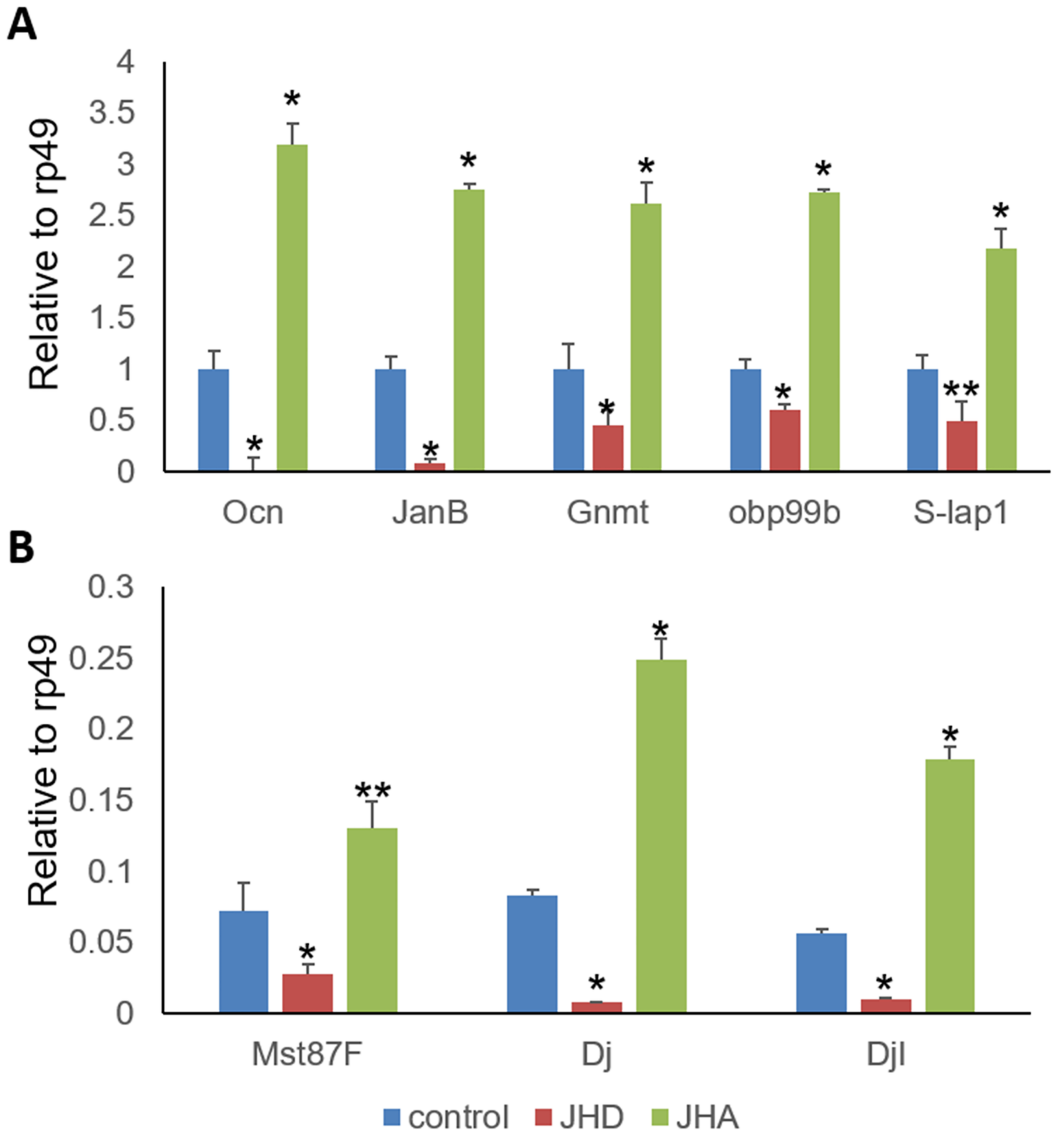
Validation of genes significantly activated by juvenile hormone analog (JHA) and significantly repressed by juvenile hormone disruptor (JHD). (A) JHD- or JHA-dependent regulation of five randomly selected JHA↑JHD↓ genes: *ocnus* (*ocn*), *janus B* (*janB*), *Glycine N-methyltransferase* (*Gnmt*), *Odorant-binding protein 99b* (*Obp99b*), and *Sperm-Leucylaminopeptidase 1* (*S-Lap1*). (B) JHD- or JHA-dependent regulation of three tTAF-dependent JHA↑JHD↓ genes: *Male-specific RNA 87F* (*Mst87F*), *don juan* (*dj*), and *don juan-like* (*djl*). Total RNA was extracted from the wandering third-instar larvae that were fed ethanol (control)-, methoprene (JHA)-, or methyl lucidone (JHD)-supplemented diet and analyzed using qPCR. Values and error bars indicate means ± SD (n = 3). *, p < 0.01 and **, p < 0.05 (*t*-test).

To validate the JHA↑JHD↓ genes, five highly abundant genes and three tTAF (testis-specific TATA-binding protein-associated factor)-dependent genes were selected from the group and further examined using quantitative RT-PCR (qPCR). The qPCR results indicated that the expression of the genes was significantly activated by JHA and significantly repressed by JHD (Fig 10). Even though these genes were testis-specific genes and, thus, upregulated in the male testis tissue, these results indicate that the genes are also expressed in the late third-instar larvae, and are regulated by JH, but show opposite effects due to JHD and JHA.

## Discussion

In the present study, JHA (methoprene)-fed larvae developed as pupae but failed to eclose, as reported previously [21], whereas JHD-fed larvae consistently died during larval and pupal development (Fig 3). The JHD phenotype differed from that of Met/GCE double deficiency, which disrupted larval-pupal transition [33]. The discrepancy between the JHD and Met/GCE double deficiency phenotypes may indicate that JHD, in addition to disrupting the heterodimer formation of JH-mediated receptor complexes, plays other roles in the disruption of JH-mediated larval development or that Met or GCE, in addition to contributing to JH-mediated gene regulation, play other roles, such as regulating caspase-dependent programmed cell death [15]. We observed the methyl lucidone or *L*. *erythrocarpa* extract interfere with the development of *Drosophila* (Fig 3). As a key observation of possible mechanisms to trigger developmental defects, we demonstrated that the JHD compound, methyl lucidone isolated from *L*. *erythrocarpa*, has effects opposing that of the JHA compound, methoprene, on the transcriptional regulation of JH-dependent genes. This may indicate that the JHD compound interferes with normal JH-dependent development by interrupting the JH-mediated gene regulation. We still could not specify the key gene groups that affect *Drosophila* development under the influence of the JHD compound. The detailed mechanism of JHD action will be addressed in further studies.

Previous studies have reported that JH promotes the synthesis of accessory gland proteins [9, 34] and that the JHA hydroprene activates the expression of MAC proteins in the red flour beetle, *T*. *castaneum* [11]. The findings of this study that JHA and JHD have opposite effects on testis-specific genes suggest that JH is also involved in the development of larval testis. In *D*. *melanogaster*, some TAF homologs are solely expressed in male testis: *Cannonball* (*Can*; dTAF5 homolog), *No hitter* (*Nht*; dTAF4 homolog), *Meiosis I arrest* (*Mia*, dTAF6 homolog), *Spermatocyte arrest* (*Sa*; dTAF8 homolog), and *Ryan express* (*Rye*; dTAF12 homolog) [35, 36]. Mutants of tTAF genes exhibit significant downregulation of spermatid differentiation-related genes, such as *Male-specific RNA 87F* (*Mst87F*), *don juan* (*dj*), *don juan-like* (*djl*), and *fuzzy onions* (*fzo*) [36–38]. Our RNA-seq test indicated that three of four tTAF-dependent genes (*Mst87F*, *dj*, and *dj-like*) and three tTAF genes (*can*, *Nht*, and *Rye*) belong to the JHA↑JHD↓ DIG group (S11 Table), which suggests that JH is involved in tTAF-related regulation during larval testis development.

**Fig 11:**
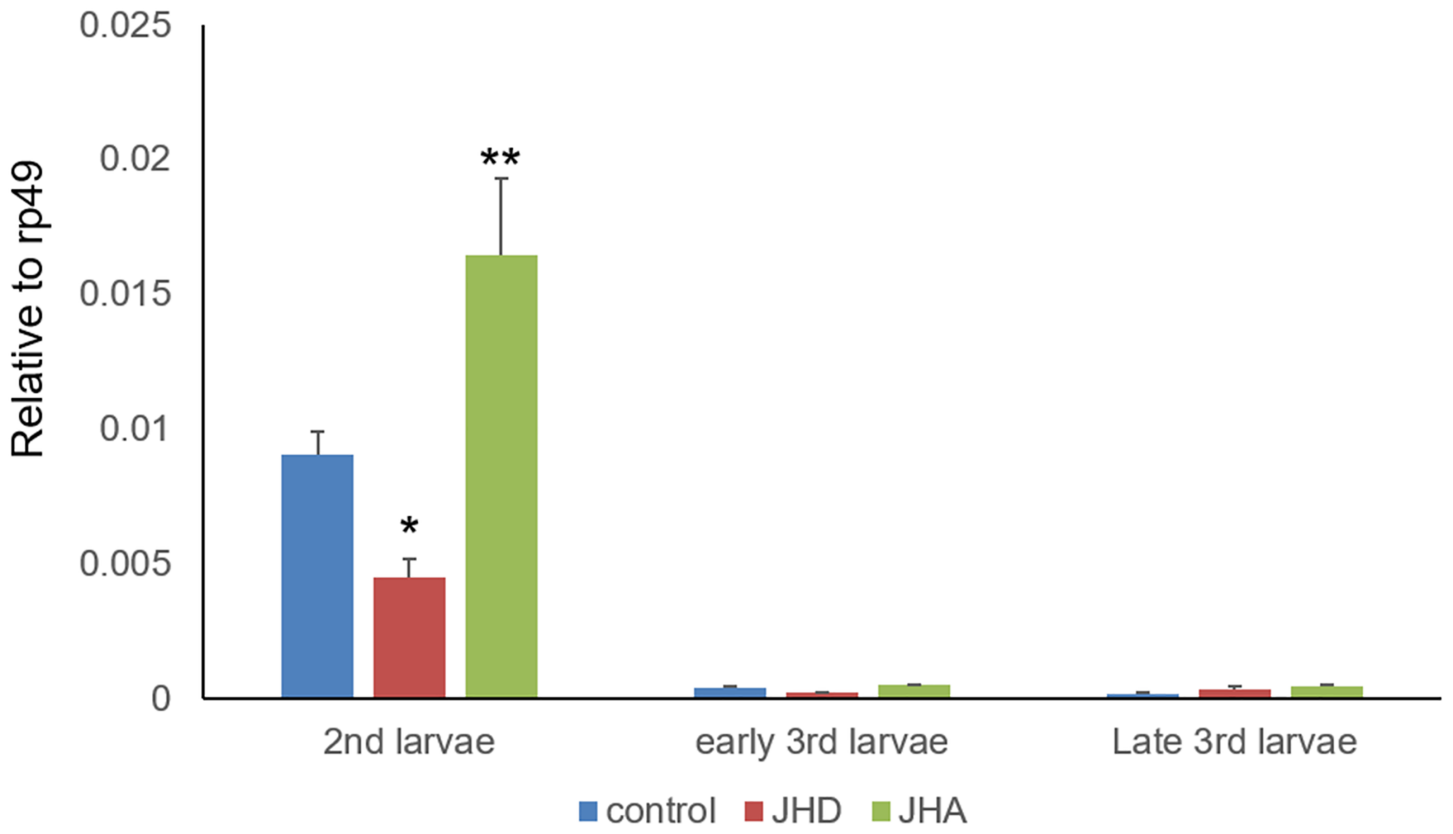
Stage-specific regulation of *Krüppel homolog 1* (*Kr-h1*) by juvenile hormone analog (JHA) and disruptor (JHD). Total RNA samples were prepared from second-, early third-, and wandering third-instar larvae fed ethanol (control)-, methoprene (JHA)-, or methyl lucidone (JHD)-supplemented diets and analyzed using qPCR. Values and error bars indicate means ± SD (n = 3). *, p < 0.01 and **, p < 0.05 (*t*-test).

The early JH-inducible gene *Krüppel-homolog 1* (*Kr-h1*) is activated by the JH/Met/SRC complex [23, 39], which plays a key role in the repression of insect metamorphosis [40–42]. As *Kr-h1* was not included in any of the four DIG groups (S1 Table), we examined the expression of *Kr-h1* in JHA- and JHD-treated larvae. The expression level of *Kr-h1* in both, the early and wandering third-instar larvae was relatively low when compared with that of second-instar larvae (Fig 11). Indeed, *Kr-h1* expression was significantly activated by JHA and significantly repressed by JHD in the second-instar larvae (Fig 11).

Both the Met and GCE proteins bind JH with high affinity at a ligand-binding pocket [17], whereas JH induces Met to bind SRC (Taiman in *Drosophila*), and the resulting JH/Met/SRC complex binds to JH-responsive DNA motifs, thereby activating the transcription of downstream target genes. Even though both GCE and Taiman are involved in the transcriptional activation of a reporter gene harboring the JH-response elements in its promoter [17], the presence of the JH/GCE/Taiman complex has not yet been reported. Our finding that GCE-Taiman binding is constitutive and is not enhanced by JH might indicate that the JH/GCE/Taiman complex is lacking in *Drosophila*. Furthermore, JH does not affect GCE-Taiman binding, and JHD disrupts GCE-Taiman binding, which suggests that JH and JHD do not directly compete for the same ligand-binding pocket of the GCE-Taiman complex and that, in this case, plant JHDs are non-structural antagonists of JH.

## Supporting information

S1 Table Transcript abundance and significance of 10,000 protein-coding genes expressed in *D*. *melanogaster* late third-instar larvae. (XLSX)

S2 Table Genes significantly activated by juvenile hormone analog (JHA) and significantly repressed by juvenile hormone disruptor (JHD). (XLSX)

S3 Table Genes significantly repressed by juvenile hormone analog (JHA) and significantly activated by juvenile hormone disruptor (JHD). (XLSX)

S4 Table Genes significantly activated by both juvenile hormone analog (JHA) and disruptor (JHD). (XLSX)

S5 Table Genes significantly repressed by both juvenile hormone analog (JHA) and disruptor (JHD). (XLSX)

S6 Table Overrepresented gene ontology groups among the JHA↑JHD↓ genes. (XLSX)

S7 Table Overrepresented gene ontology groups among the JHA↓JHD↑ genes. (XLSX)

S8 Table Overrepresented gene ontology groups among the JHA↑JHD↑ genes. (XLSX)

S9 Table Overrepresented gene ontology groups among the JHA↓JHD↓ genes. (XLSX)

S10 Table RNA-seq analysis of juvenile hormone analog (JHA)- and disruptor (JHD)-dependent regulation of tTAF and tTAF-dependent genes. *, the expression of these genes was significantly affected by both JHA and JHD but not enough to be included in S2 Table.
(PDF)

S11 Table Summary of RNA-seq experiments. RNA-seq libraries were constructed from poly(A)-RNA extracted from 10 wandering third-instar *D*. *melanogaster* larvae fed either ethanol (control)-, methoprene (juvenile hormone analog)-, or methyl lucidone (juvenile hormone disruptor)-supplemented diet. Values indicate the number and quality of reads from each library.
(PDF)
