# Supplementary material for: A plant diterpene counteracts juvenile hormone-mediated gene regulation during Drosophila melanogaster larval development
Source: PLoS One. 2018 Jul 16;13(7):e0200706. doi: 10.1371/journal.pone.0200706 (PMC6047816; doi:10.1371/journal.pone.0200706)
Supplement: S10 Table — *, the expression of these genes was significantly affected by both JHA and JHD but not enough to be included in S2 Table. (PDF) [file pone.0200706.s010.pdf]

Gene	gene definition/molecular function	JH-dependent expression*
can	TAF5	JHA↑JHD↓*
mia	TAF6	no
sa	TAF8	no
nht	TAF4	JHA↑JHD↓*
Taf12L (rye)	TAF12	JHA↑JHD↓
Mst87F	tTAF-dependent	JHA↑JHD↓
dj	tTAF-dependent, don juan	JHA↑JHD↓
djl	tTAF-dependent, don juan like	JHA↑JHD↓
fzo	tTAF-dependent	no
