# Supplementary material for: A plant diterpene counteracts juvenile hormone-mediated gene regulation during Drosophila melanogaster larval development
Source: PLoS One. 2018 Jul 16;13(7):e0200706. doi: 10.1371/journal.pone.0200706 (PMC6047816; doi:10.1371/journal.pone.0200706)
Supplement: S11 Table — RNA-seq libraries were constructed from poly(A)-RNA extracted from 10 wandering third-instar D. melanogaster larvae fed either ethanol (control)-, methoprene (juvenile hormone analog)-, or methyl lucidone (juvenile hormone disruptor)-supplemented diet. Values indicate the number and quality of reads from each library. (PDF) [file pone.0200706.s011.pdf]

Sample ID	Read length	# Reads	% of >= Q20 Bases
Control 1	101 bp	117,382,985	94.5
Control 2	101 bp	97,752,698	94.67
Control 3	101 bp	79,324,454	98.056
JHA 1	101 bp	103,263,226	94.63
JHA 2	101 bp	101,751,544	94.68
JHA 3	101 bp	83,191,174	98.153
JHD 1	101 bp	120,940,581	94.7
JHD 2	101 bp	73,883,137	98.079
JHD 3	101 bp	80,675,844	98.083
